# 26. Is There a Correlation Between Reactogenicity and Immune Responses of the Adjuvanted Recombinant Zoster Vaccine (RZV)? A Post-hoc Analysis

**DOI:** 10.1093/ofid/ofab466.228

**Published:** 2021-12-04

**Authors:** Andrea Callegaro, David O Willer, Wivine Burny, Caroline Hervé, Joon Hyung Kim, myron J levin, Toufik Zahaf, Anthony L Cunningham, Arnaud Didierlaurent

**Affiliations:** 1 GSK, Rixensart/Wavre, Belgium, Rixensart/Wavre, Brabant Wallon, Belgium; 2 GSK, Markham, ON, Canada; 3 GSK, RIxensart/Wavre, Belgium, braine-l’alleud, Brabant Wallon, Belgium; 4 University of Colorado Anschutz Medical Campus, Aurora, Colorado; 5 The Westmead Institute for Medical Research and the Institute’s Centre for Virus Research, The University of Sydney, Sidney, New South Wales, Australia

## Abstract

**Background:**

RZV (GSK) contains the varicella-zoster virus antigen glycoprotein E (gE) and the adjuvant system AS01_B_ that enhances gE-specific immune responses through stimulating innate immunity. AS01_B_ may contribute to the development of transient local or systemic post-vaccination reactions. A hypothesis that the magnitude of those reactions is predictive of immunogenicity and efficacy (i.e., “no pain, no gain”) remains untested. To evaluate potential correlations between RZV’s reactogenicity and immunogenicity in adults aged ≥ 50 years, a *post-hoc* analysis was conducted using data from 2 large phase 3 studies (NCT01165177, NCT01165229).

**Methods:**

Reactogenicity was calculated as a single score per symptom (maximum grade recorded over 7 days post-vaccination). A global score obtained by adding each maximum severity for all reported symptoms (multivariate reactogenicity models) and a score for each reactogenicity symptom (univariate reactogenicity models) were estimated.

**Results:**

The analysis included 904 and 147 RZV recipients with completed post-vaccination symptom diary cards and with anti-gE antibody results or cell-mediated immunity (CMI) results, respectively. The global score of reactogenicity post-dose 2 was significantly associated with anti-gE antibody response (p< 0.001, estimate 0.112) although the absolute antibody increase associated with reactogenicity was minimal (1.29-fold increase), while the association with CMI response was not statistically significant (p=0.073, estimate 0.230). There was a weak, but statistically significant association between gE-specific immune responses and the maximum pain post-dose 2 score (p=0.001, estimate 0.041), irrespective of post-vaccination time. Nevertheless, there are observations of immune responses in participants for whom pain was not reported.

**Conclusion:**

A weak but statistically significant correlation was found between injection site pain intensity and immune responses in adult RZV recipients aged ≥ 50 years. However, participants reporting no pain were also able to mount a strong immune response, therefore pain cannot be a surrogate marker to inform on the level of immune response or on likelihood of being protected against herpes zoster.

**Funding:**

GlaxoSmithKline Biologicals SA

**Disclosures:**

**Andrea Callegaro, PhD**, **GSK group of companies** (Employee, Shareholder) **David O. Willer, PhD**, **GSK group of companies** (Employee, Other Financial or Material Support, Receive GSK shares as part of employment renumeration) **Wivine Burny, PhD**, **GSK group of companies** (Employee) **Caroline Hervé, PhD**, **GSK group of companies** (Employee) **Joon Hyung Kim, MD**, **GSK group of companies** (Employee, Shareholder) **myron J. levin, MD**, **GSK group of companies** (Employee, Research Grant or Support) **Toufik Zahaf, PhD**, **GSK group of companies** (Employee, Shareholder) **Anthony L. Cunningham, F.A.H.M.S., MD, M.B.B.S., B. Med. Sci. (Hons), F.R.A.C.P., F.R.C.P.A., F.A.S.M.**, **GSK group of companies** (Grant/Research Support, Advisor or Review Panel member, Speaker’s Bureau) **Arnaud Didierlaurent, PhD**, **GSK group of companies** (Other Financial or Material Support, previous employee until 03/2020)**Sanofi** (Speaker’s Bureau)

